# The Surgical Management of Pilonidal Disease is Uncertain Because of High Recurrence Rates

**DOI:** 10.7759/cureus.2625

**Published:** 2018-05-14

**Authors:** David Burnett, Stephen R Smith, Christopher J Young

**Affiliations:** 1 Department of Colorectal Surgery, John Hunter Hospital, New Lambton Heights, AUS; 2 Colorectal Unit, Royal Prince Alfred Hospital

**Keywords:** pilonidal disease, sacrococcyceal pilonidal sinus disease

## Abstract

Background

Pilonidal disease is a common condition with no consensus for the best management of chronic disease or current practice in Australia and New Zealand.

Methods

A survey was distributed among 190 colorectal and 592 general surgeons in Australia and New Zealand. Data was obtained regarding pilonidal surgery volume, procedures performed, non-operative management and recurrence rates. Three clinical scenarios were also presented.

Results

The response rate was 58% among colorectal surgeons, 18% among general surgeons. Nineteen percent of surgeons were high-volume (>23 operations per year), 47% low-volume (<12 operations per year). The commonest procedure was the Karydakis procedure (77%), with many others performed including rhomboid flaps (36%), Bascom cleft lift (13%), Z-plasty (7%), and gluteal rotation flaps (5%). Fifty-five percent of high-volume surgeons offered more than one operation while only 16% of low-volume surgeons did. Nineteen percent operated on all patients with pilonidal disease, 89% believing off-midline closure to be superior to midline. Disease extent was the main driver for non-operative management; patient factors such as cosmesis and time-off work being the least important. Sixty-four percent reported recurrence rates above 5%, and 37% recurrence rates >10%. Six percent reported no recurrences ever. Five percent reported recurrence rates over 20%, but 24% stated that over one-fifth of their practice consists of recurrent disease.

Conclusions

This study reports higher recurrence rates than in published series, suggesting many surgeons do not see their own recurrences, with current treatment not as successful as previously thought. Combined with the widespread variation in practice, optimal management of this disease remains unclear.

## Introduction

Pilonidal disease is a common condition with an estimated population incidence of 26 per 100,000 in Australia and New Zealand (ANZ) [1]. The disease usually manifests itself in the second and third decades. Males are more commonly affected and risk factors include family history, obesity, hirsutism and a sedentary lifestyle [1,2]. The aetiology is poorly understood with theories proposed by Bascom and Karydakis, with some evidence to support each [3,4].

There is little controversy about the treatment of an acute pilonidal abscess; off-midline incision and drainage is associated with the fastest healing time and lowest recurrence rate [5,6]. But the management of chronic or recurrent disease is less uniform with many surgical options and varying success rates published [4,7-9]. The dissatisfaction with all methods of surgical treatment has long been discussed [10]. The publication in 1992 by Karydakis of a large surgical series of pilonidal sinus with low recurrence rate had a significant practice impact on ANZ surgical practice [4]. Kitchen described this procedure in somewhat more detail in 1996, only enhancing its understanding and reputation [7]. There certainly is support for the benefits of off-midline natal cleft closure techniques for primary closure of chronic pilonidal sinus compared with simple midline closure [11]. A Cochrane review from 2007 did not show any benefit of open healing compared with primary closure of pilonidal sinus surgery wounds [12]. Published practice parameters even highlight that evidence for surgical procedures for primary, complex and recurrent pilonidal disease is mostly only of moderate-quality [13]. Even data from just some of the literature published this year reveals the consternation this disease causes and the heterogeneous approaches to treatment [14-18]. The wide range of options being offered cannot be due to the great success of any one of them, but rather the frustration with the imperfect results of all of them.

The aim of this survey was to assess the current management of pilonidal disease in the elective setting in Australia and New Zealand.

## Materials and methods

Ethical approval for this study was obtained from the Hunter New England Human Research Ethics Committee (HNEHREC: 13/10/16/5.05), with subsequent approval by the research committees of the Colorectal Surgical Society of Australia and New Zealand (CSSANZ) and General Surgeons Australia (GSA).

Between March and October 2014, an online questionnaire was distributed to the 190 members of CSSANZ, and the 592 members of GSA who were also not CSSANZ fellows.

The survey consisted of 21 questions, with respondents asked to provide information about their volume of pilonidal surgery, non-operative management, procedures performed and recurrence rates. Respondents were also given three clinical scenarios and asked for their recommended approach. Scenario one involved a 30-year-old male plumber, concerned about time off work, who had undergone previous pilonidal surgery and presented with a single discharging midline pit. Scenario two involved a 16-year-old male who had undergone six previous operations and had a recurrent disease with a 1 cm sinus in the natal cleft. Scenario three involved a 19-year-old female with fair skin and dark hair who had undergone a previous abscess drainage and now had swelling in the natal cleft. She was extremely worried about cosmesis.

Survey respondents were grouped into high-volume (>24 procedures yearly), moderate-volume (12-23 procedures yearly) and low-volume surgeons (<12 procedures yearly). Statistical analyses were performed using Excel.

## Results

There were 111 responses from 190 colorectal surgeons (58% response rate), and 105 of 592 (18%) general surgeons, a total response rate of 216 (28%). Eighteen general surgeons answered that pilonidal surgery was not part of their practice and were excluded. A further six answered no questions at all, leaving 192 responses available for analysis. As not every surgeon answered every question, the different number of respondents for each question is denoted by the ‘n’ value. Surgeon distribution by volume group was 37 (19%) high-volume, 64 (33%) moderate-volume and 91 (47%) low-volume surgeons, with more colorectal surgeons in the high and moderate-volume groups (Table 1).

**Table 1:** Surgeon distribution by specialty and pilonidal surgery volume.

Surgeon volume groups	Number of procedures per year	Colorectal surgeons (n)	General surgeons (n)	Total (n)
High volume	>23	25	12	37
Moderate volume	12-23	40	24	64
Low volume	0-11	44	47	91
Total		109	83	192

Thirty-five out of 187 respondents (19%) operate on all patients with pilonidal disease and 20 surgeons (10%) operate on less than half. The percentage of patients managed non-operatively was similar across the high, moderate, and low-volume groups (Table 2).

**Table 2:** Distribution of patients with pilonidal disease undergoing surgery according to surgeon yearly volume groups.

	Percentage of a surgeon’s patients undergoing surgery									
Surgeon volume groups	100%		90-99%		80-89%		50-79%		<50%	
	n	%	n	%	N	%	n	%	n	%
High volume (n = 36)	6	17	12	33	4	11	11	31	3	9
Moderate volume (n = 63)	10	16	20	32	9	14	15	24	9	14
Low volume (n = 88)	19	22	19	22	21	24	21	24	8	9
Total (n = 187)	35	19	51	27	34	18	47	25	20	11

**Figure 1:**
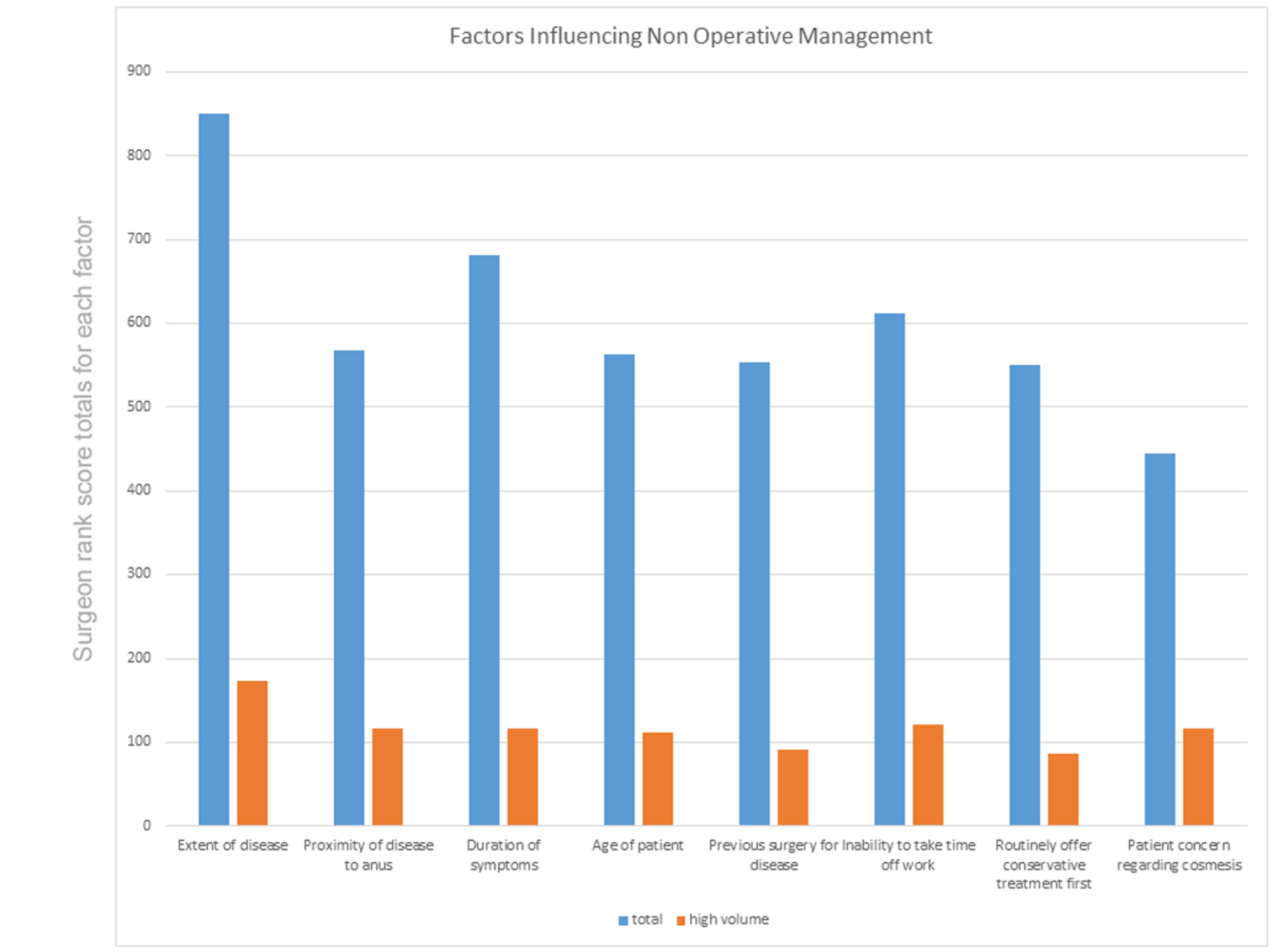
Surgeon responses to factors influencing non-operative management of pilonidal disease according to all surgeons and high volume surgeons.

A total of 134 respondents ranked eight factors suggested as most likely to influence their decision not to operate. These factors were scored by each surgeon from one to eight (least likely to most likely, thereby distributing 36 possible rank scores per surgeon across the eight factors) and the resulting ranks for each factor were added together to give a total measure of importance (Figure 1).

For all surgeons, disease extent was the main driver to manage non-operatively, patient cosmesis being the least important. Proximity to the anus is more important among high-volume surgeons.

Eighty-nine percent (163/183) of respondents believe an off-midline closure is superior to a midline wound. Fifteen percent (13/86) of low-volume surgeons, 7% (4/61) of moderate-volume and 9% (3/35) of high-volume surgeons believe that midline closure is as good as a lateral wound.

Almost half the surgeons surveyed, 84 out of 183 (46%), do not believe that routine hair removal following surgery reduces the risk of recurrence. Despite this, the majority of respondents (71%) advise hair removal as an adjunct to surgery. Methods recommended included shaving (61%), laser (47%), depilatory agents (37%), and waxing (33%).

Fifty-five percent of high-volume surgeons perform more than one operation while only 16% of low-volume surgeons do. The most common ‘off-midline’ operation across all groups is the Karydakis or modified Karydakis procedure, with 126 respondents (77%) undertaking this procedure. Rhomboid flap surgery is more common among high-volume surgeons (55%) compared to moderate (36%) and low-volume surgeons (27%). Gluteal rotation flaps, usually reserved for extensive disease, are performed by only 5% of respondents and mainly by high-volume surgeons (Table 3).

**Table 3:** Pilonidal procedures performed according to surgeon yearly volume groups.

Surgeon volume groups	Karydakis		Rhomboid flap		Bascom cleft lift		Z plasty		Gluteal rotation flap		More than one procedure		
	n	%	n	%	n	%	n	%	n	%	n	%	
High (n = 31)	25	80	17	55	5	16	1	3	3	10	17	55	
Moderate (n = 58)	45	78	21	36	11	9	5	9	1	2	22	38	
Low (n = 74)	56	76	20	27	6	8	6	8	4	5	12	16	
Total (n = 163)	126	77	58	36	22	13	12	7	8	5	51	31	

Respondents were asked to estimate their recurrence rates after pilonidal surgery. Of the 184 surgeons who answered this question, 50% estimated a recurrence rate of between 1% and 10%, and another 32% quoted recurrence rates of 10-20% (Table 4).

**Table 4:** Surgeons’ own estimated recurrence rates according to surgeon yearly volume groups and specialty. CSSANZ: Colorectal Surgical Society of Australia and New Zealand; GSA: General Surgeons Australia.

Recurrence rate	<1%		1-5%		5-10%		10-20%		>20%	
	n	%	n	%	n	%	n	%	n	%
High volume (n = 34)	1	3	13	38	9	26	10	29	1	3
Moderate volume (n = 62)	9	15	12	19	16	26	22	36	3	5
Low volume (n = 87)	13	15	19	22	22	25	27	31	6	7
CSSANZ member (n = 104)	8	8	23	22	33	32	33	32	7	7
GSA member (n = 72)	14	19	19	26	15	21	22	31	2	3
Total (n = 184)	23	13	44	24	48	26	59	32	10	5

Thirteen percent of respondents estimated a recurrence rate of <1%, including 6% who reported never having had a recurrence. There were more moderate and low-volume surgeons (15%) reporting recurrence rates of <1%, compared to high-volume surgeons (3%).

Overall the reported recurrence rates were similar between colorectal surgeons and general surgeons, with the exception of 19% of general surgeons reporting recurrence rates of <1% compared to only 8% of CSSANZ members.

Surgeons were also asked to estimate what proportion of their pilonidal practice consists of recurrences in general, and what proportion are recurrences from other surgeons specifically (Table 5).

**Table 5:** Surgeons’ reporting of recurrent disease encountered in their practice.

Recurrence rate	<1%		1-5%		5-10%		10-20%		>20%	
	n	%	n	%	N	%	n	%	n	%
Own recurrence (n = 184)	23	13	44	24	48	26	59	32	10	5
Proportion of practice is recurrent disease (n = 193)	19	10	39	20	44	23	46	24	45	3
Proportion of practice is recurrent disease from other surgeons (n = 188)	30	16	35	19	42	22	36	19	45	24

Interestingly, over 20% of surgeons reported more than 20% of their practice involved recurrences, compared to 5% reporting their own recurrence rate at >20%.

When presented with the scenario of a minimally symptomatic recurrence in a young man who cannot afford to take much time off work, only 16% of surgeons recommended non-operative management (Table 6).

**Table 6:** Surgeons’ answers to three scenarios (please refer text for scenario details).

	Scenario 1		Scenario 2		Scenario 3	
Recommendation	n	%	n	%	n	%
Conservative/hair removal	28	16	12	7	25	14
Cleaning/Curettage tracts	20	11	16	9	14	8
Bascom’s type 1 procedure	11	6	2	1	7	4
Lay open +/- marsupialisation	20	11	30	17	25	14
Excision and primary closure	20	11	2	1	30	17
Bascom’s cleft procedure	4	2	6	3	4	2
Karydakis procedure	51	29	39	22	50	28
Rhomboid flap	15	8	39	22	7	4
Z-plasty flap	2	1	6	3	0	0
Other	7	4	25	14	16	9
Total	178	100	177	100	178	100

The majority of respondents (55%) recommended a Karydakis procedure.

The second scenario of a young man who has undergone six previous operations and now has a chronic sinus triggered a wide variety of approaches (Table 6), most commonly a rhomboid flap (22%) or Karydakis procedure (22%). Fifteen percent suggested other options, the majority of these were to refer to a plastic surgeon (48%) or excise and apply a VAC dressing (32%).

In the third scenario of a 19-year-old female with concerns about cosmesis, respondents again chose considerably different options (Table 6). As in the previous scenarios, the most common response was to perform a Karydakis procedure (28%), followed by excision and primary closure (17%). A higher proportion of respondents (14%) recommended non-operative management in this scenario compared to the previous ones.

## Discussion

This survey confirms that pilonidal disease is seen and managed by both colorectal and general surgeons. Most high-volume surgeons are members of the CSSANZ but over 40% of respondents to this survey were general surgeons, and both groups see and treat a reasonably large proportion of recurrent disease.

The incidence of pilonidal disease in the young adult population is common. Time off normal activities following surgery, together with a significant recurrence rate, mean there is a substantial socio-economic burden associated with this disease [1,2].

Despite its natural history and the complications associated with surgery almost one in five respondents recommend an operation for all presentations. The morbidity and socioeconomic impact of this approach may not be justified, especially in minimally symptomatic patients.

Patient concerns such as time off work and cosmesis were less important in decision making than extent of disease. Surprisingly, proximity to the anus was one of the least important factors, although in the high-volume group it became more important. One explanation is that high-volume surgeons are more likely to see extensive disease close to the anus and therefore this consideration bears more weight in their planning compared to surgeons who see predominantly superior natal cleft disease.

Despite reasonable evidence supporting off-midline closure [5], 11% of surgeons treating pilonidal disease still believe a midline wound is non-inferior. This includes 9% of high-volume surgeons. Although almost half of the respondents do not believe that removal of hair affects recurrence of disease, the majority still recommend hair removal by shaving, despite evidence demonstrating an increased recurrence rate in patients who shave [19].

By far the most common operation undertaken for pilonidal disease is a Karydakis or modified Karydakis procedure, performed by almost 80% of respondents. The initial series published by Karydakis reported a complication rate of 8.5% with a recurrence rate of <1% [4]. Kitchen reported a Karydakis procedure series with similar complication rates and a recurrence rate of 4% [7].

In this study, almost 40% of surgeons estimated their own recurrence rates to be over 10%. As the most common operation is the Karydakis, this suggests the recurrence rate outside of clinical trials and published series may be considerably higher than previously thought. The results of this survey suggest that although high-volume surgeons tailor their approach to pilonidal disease, increased pilonidal disease surgical volume does not translate into a lower recurrence rate. One explanation is that higher volume surgeons are more likely to see extensive disease, necessitating more complex surgery and carrying a higher risk of recurrence.

The three scenarios included in this survey serve to illustrate the breadth of procedures undertaken for pilonidal disease. The Karydakis procedure was the most common recommended treatment in all three scenarios. This may be due to lack of familiarity with other techniques, or a belief that this procedure offers the best outcome. We note that in the third scenario of a 19-year-old female, the second most common response was excision and primary closure (17%), despite its known high recurrence rate [6].

A limitation of this study is the low response rate from general surgeons. One possible explanation is that many general surgeons practise in specialties where they do not see pilonidal disease and did not respond to the survey request. The higher response rate from colorectal surgeons supports this explanation.

## Conclusions

The responses from this survey suggest underreporting of recurrence for pilonidal disease surgery. Although 23% of surgeons had a practice exceeding 20% recurrent disease, only 5% of respondents felt their own recurrence rate exceeded 20%. This suggests that surgeons often do not see or hear about their own recurrences, and that recurrence rates from published series may be less than actual rates.
